# Potential prognostic marker ubiquitin carboxyl-terminal hydrolase-L1 does not predict patient survival in non-small cell lung carcinoma

**DOI:** 10.1186/1756-9966-30-79

**Published:** 2011-08-30

**Authors:** Katy S Orr, Zhanzhong Shi, W Mark Brown, Kathleen A O'Hagan, Terence R Lappin, Perry Maxwell, Melanie J Percy

**Affiliations:** 1 Department of Haematology, Centre for Cancer Research and Cell Biology, Queen's University Belfast, 97 Lisburn Road, Belfast, Northern Ireland, UK, BT9 7BL

## Abstract

**Background:**

Ubiquitin Carboxyl-Terminal Hydrolase-L1 (UCH-L1) is a deubiquitinating enzyme that is highly expressed throughout the central and peripheral nervous system and in cells of the diffuse neuroendocrine system. Aberrant function of UCH-L1 has been associated with neurological disorders such as Parkinson's disease and Alzheimer's disease. Moreover, UCH-L1 exhibits a variable expression pattern in cancer, acting either as a tumour suppressor or promoter, depending on the type of cancer. In non-small cell lung carcinoma primary tumour samples, UCH-L1 is highly expressed and is associated with an advanced tumour stage. This suggests UCH-L1 may be involved in oncogenic transformation and tumour invasion in NSCLC. However, the functional significance of UCH-L1 in the progression of NSCLC is unclear. The aim of this study was to investigate the role of UCH-L1 using NSCLC cell line models and to determine if it is clinically relevant as a prognostic marker for advanced stage disease.

**Methods:**

UCH-L1 expression in NSCLC cell lines H838 and H157 was modulated by siRNA-knockdown, and the phenotypic changes were assessed by flow cytometry, haematoxylin & eosin (H&E) staining and poly (ADP-ribose) polymerase (PARP) cleavage. Metastatic potential was measured by the presence of phosphorylated myosin light chain (MLC2). Tumour microarrays were examined immunohistochemically for UCH-L1 expression. Kaplan-Meier curves were generated using UCH-L1 expression levels and patient survival data extracted from Gene Expression Omnibus data files.

**Results:**

Expression of UCH-L1 was decreased by siRNA in both cell lines, resulting in increased cell death in H838 adenocarcinoma cells but not in the H157 squamous cell line. However, metastatic potential was reduced in H157 cells. Immunohistochemical staining of UCH-L1 in patient tumours confirmed it was preferentially expressed in squamous cell carcinoma rather than adenocarcinoma. However the Kaplan-Meier curves generated showed no correlation between UCH-L1 expression levels and patient outcome.

**Conclusions:**

Although UCH-L1 appears to be involved in carcinogenic processes in NSCLC cell lines, the absence of correlation with patient survival indicates that caution is required in the use of UCH-L1 as a potential prognostic marker for advanced stage and metastasis in lung carcinoma.

## Background

Ubiquitination is a highly diverse and complex post-translational modification responsible for controlling protein expression and activity in a vast array of cellular processes such as proteasomal degradation, cell cycle regulation, protein trafficking, inflammation and DNA repair [1,2]. Removal of ubiquitin via the action of deubiquitinating enzymes (DUBs) is integral to the regulation of the ubiquitin system, hence the importance of these enzymes in the maintenance of protein expression and function. There are 5 classes of DUBs and Ubiquitin Carboxyl Terminal Hydrolase-L1 (UCH-L1), a member of the UCH family, catalyses the hydrolysis of ubiquitin from ubiquitin precursors and from ubiquitinated products following proteasomal degradation of polyubiquitinated proteins [3-6]. Therefore UCH-L1 is responsible for conserving the cellular pool of ubiquitin and it has also been implicated in cellular pathways such as proliferation, apoptosis and cell migration [7]. A unique characteristic of UCH-L1 is its ability to act as an ubiquitin ligase in dimeric form, in contrast to acting as a hydrolase in its monomeric form [8].

UCHL-1 is highly expressed in the central and peripheral nervous system, reproductive tissue and neuroendocrine (NE) cells, although it is expressed in most adult tissues [9,10]. In both reproductive organs and nervous tissue, UCH-L1 promotes apoptosis. In testicular germ cells UCH-L1 expression is responsible for an early apoptotic wave during spermatogenesis but tight regulation of UCH-L1 is important as high levels cause excessive apoptosis in the ovaries and testes of transgenic mice [5,11]. In retinal neurons the regulation of intracellular ubiquitin by UCH-L1 alters the stability of pro-apoptotic and anti-apoptotic proteins with a substantial increase in Bcl-2 and XIAP levels in UCH-L1 null mice compared to UCH-L1 wildtype [12,13]. Aberrant UCH-L1 function in neurons manifests as neurological diseases, such as Parkinson's disease (PD), where dysfunctions of the ubiquitin-proteasome system allow the accumulation of α-synuclein, which is important in the pathology of the disease. Mutations in *UCH-L1 *have been detected in cases of familial PD. In particular the I93 M amino-acid substitution has been linked to a rare inherited form of PD known as PARK5 [5,14], whereas the S18Y polymorphism reduces susceptibility to PD [15].

In cancer, UCH-L1 exhibits highly variable expression patterns seemingly in a tumor-specific manner. UCH-L1 can act as a tumor-suppressor and is silenced in ovarian [16], hepatocellular [9,17], renal cell [17,18], head and neck [19] and oesophageal carcinomas [20], when compared to normal tissue. The silencing in many cases is due to hypermethylation of the *UCH-L1 *promoter region [16,20-22]. On the contrary, UCH-L1 is over-expressed in neuroblastoma [23], lung carcinoma, independent of neuronal differentiation [24], myeloma [25], prostate carcinoma [26], osteosarcoma [27] and pancreatic carcinoma [28]. Several types of cancer present contradictory results in relation to UCH-L1 expression patterns and this is the case in both colorectal and breast carcinoma [16,29-31].

In non-small cell lung carcinoma (NSCLC) UCH-L1 is consistently highly expressed in both cell lines and primary tumour samples when compared to normal lung tissue where the expression of UCH-L1 is confined solely to cells of the neuroendocrine (NE) system. The presence of high levels of UCH-L1 has also been associated with an advanced tumor stage suggesting a possible role of UCH-L1 in oncogenic transformation and tumor invasion in NSCLC [32,33]. A correlation has been found between UCH-L1 expression and histological type, with squamous cell carcinomas expressing the protein more frequently than adenocarcinomas [24,34].

The distinction between different types of NSCLC was until quite recently, clinically unimportant. It was necessary only to decide if a patient had NSCLC or small cell carcinoma, a determination which can be made robustly on morphology. With the development of drugs such as Pemetrexed (Alimta™), which shows more activity against non-squamous NSCLC and Bevacizumab (Avastin™), which is contraindicated for use in squamous cell carcinoma, the further classification of NSCLC type is now the clinical standard. The distinction is made on the basis of morphology, histochemistry (mucin staining with Alcian blue/Periodic acid Schiff) and immunohistochemistry for thyroid transcription factor 1 (TTF-1), cytokeratins (CK) 5/6 and p63 amongst other possible combinations. Squamous differentiation is indicated by positivity with CK5/6 and p63 whilst TTF-1 is negative [35]. Therefore, the differential expression of UCH-L1 in NSCLC has a particular relevance given this impetus for classification of tumor type.

To establish whether UCH-L1 plays an important role in the pathogenesis of lung carcinoma we used two NSCLC cell lines of different subtypes to investigate the phenotypic effects observed following silencing of UCH-L1. We found that UCH-L1 expression increases apoptotic resistance in the adenocarcinoma cell line (H838) and promotes cell migration in the H157 squamous cell carcinoma cell line. Also, in NSCLC tumor samples we showed that UCH-L1 is preferentially expressed in squamous cell carcinoma. To examine the importance of UCH-L1 in patient samples we analyzed NSCLC patient survival data but despite the oncogenic role found in the NSCLC cell lines, no correlation between UCH-L1 expression and survival was evident.

## Methods

### Cell Culture

All cell lines were maintained in RPMI 1640 medium containing 10% fetal bovine serum (PAA, Pasching, Austria), 100 U/ml penicillin and 100 μg/ml streptomycin (Invitrogen, Paisley, UK), except BEAS-2B, MPP-89 and REN cells which were maintained in GIBCO^® ^F12 (Ham) Nutrient Mixture (Invitrogen), supplemented with 10% FBS, 1% Penicillin/Streptomycin, 1% L-glutamine and 1% Non-Essential Amino Acids. The cells were grown in a humidified incubator (Sanyo, San Diego, CA) at 37°C with 5% CO_2_.

### Quantitative PCR

*UCH-L1 *mRNA expression in parental and UCH-L1 siRNA-treated H157 and H838 cells was measured by quantitative-PCR (q-PCR). Primers and probes for *UCH-L1 *(assay ID: Hs00188233_m1) and 18S RNA internal control (assay ID: Hs99999901_s1) were obtained from Applied Biosystems (Foster City, CA). Reactions were carried out on the ABI Prism 7500 system equipped with a 96-well thermal cycler as previously described [36]. Briefly, total RNA was extracted from cells with TRIzol (Invitrogen) and cDNA was obtained by reverse transcription. Data were collected and analyzed with Sequence Detector 7500 System v2.1 software (Applied Biosystems) and relative gene expression was calculated using the ΔΔCt method.

### Sequencing of UCH-L1 gene

DNA was extracted from each cell line using the DNeasy Blood and Tissue Kit (Qiagen, West Sussex, UK). PCR-directed sequencing was performed using standard protocols (primers available on request). The DNA sequencing data was viewed and analysed using Chromas Lite software (Technelysium Pty Ltd., Shannon, Ireland) and SeqMan™ II software (DNA Star, West Lothian, UK).

### Immunoblotting

Western blot analysis was used to detect the expression level of proteins as previously described [37]. Primary antibodies used were anti-UCH-L1, anti-Phospho-MLC2, anti-MLC2 (New England Biolabs, Hitchin, UK), anti-PARP (eBioscience, Hatfield, UK) and anti-β-actin (Sigma-Aldrich, Dorset, UK).

### siRNA transient transfection

UCH-L1 siRNA (synthesized by Dharmacon, Thermo Fisher Scientific, Loughborough, UK) was transiently transfected into H838 and H157 cells in 6-well plates using siPORT NeoFX transfection agent according to the manufacturer's recommendations (Ambion, Applied Biosystems). Briefly, prior to the transfection, cells were trypsinised then resuspended in media without antibiotics at a cell density of 1 × 10^5^/ml. For each transfection reaction, 5 μl of siPORT NeoFX reagent was applied to 95 μl of Opti-MEM medium (Invitrogen), incubated at room temperature for 10 min, then mixed with an equal volume of UCH-L1 siRNA solution (to give a final concentration of 10 nM). After incubation at room temperature for 10 min, the siRNA transfection complexes were dispersed into 6-well plates and overlaid by cell suspensions, gently mixed and incubated for 48 to 72 hr at 37°C, 5% CO_2_. Transfection efficiency was assessed by q-PCR and Western blot.

### Phase-contrast microscopy

Phase-contrast microscopy with a Zeiss Axiovert 200 phase-contrast microscope (Carl Zeiss Microimaging Inc., Welwyn Garden City, UK) equipped with an Orca camera (Hamamatsu Photonics, Hamamatsu City, Japan) was used to observe the morphological changes in H838 cells 48 hr post-transfection of UCH-L1 siRNA.

### Haematoxylin & eosin staining and light microscopy

Transiently transfected H838 cells were grown on coverslips. At 48 hr after transfection, the cells were fixed in 90% ethanol, stained with haematoxylin & eosin (H&E) and viewed under light microscope for signs of apoptosis. The cells with abnormal nuclear features such as a fragmented nucleus or breakdown of the nuclear membrane were classified as apoptotic. For each slide, the numbers of apoptotic cells in 20 different fields at 250× magnification were counted.

### Flow Cytometry

At 72 hr post-transfection cells were harvested by trypsinisation and fixed by ice-cold 70% ethanol for 1 hr. The fixed cells were washed twice with PBS and stained with 0.5 ml of 40 μg/ml propidium iodide (PI) at 37°C for 30 min protected from light. The PI-stained samples were analyzed by the BD™ LSR II FACS instrument and the BD™ FACS Flow Supply System (BD Biosciences, CA) and a total of 10,000 events were analyzed. The hypodiploid sub-population in sub-G1/G0 phase was regarded as apoptotic cells and the percentages of these cells were calculated using the BD™ FACS Diva software v.6.1.2.

### Immunohistochemistry of cell lines and patient samples

Formalin-fixed paraffin wax-embedded cell blocks of H157, H838 and BEAS-2B cells and paraffin wax embedded sections from 140 samples of NSCLC were stained for UCHL-1 expression. Briefly, sections were pre-treated in a 750 W microwave oven (0.1 M citrate buffer, pH 6.0) for 22 minutes, cooled rapidly, washed in Tris-buffered Saline and were incubated in mouse anti-UCHL-1 (NCL-PGP9.5, 1:100; Novocastra, Newcastle Upon Tyne, UK) overnight at 4°C. Localisation was achieved using Envision peroxidise kit as recommended by the manufacturer (Dako, Ely, UK). All sections were counterstained in Meyer's haematoxylin. Immunoreactivity was assessed by two observers and percentage positive agreed. A cut-off value of 10% was used for UCH-L1 results. Selected sections were incubated with mouse immunoglobulin as negative controls. All tissues were used under regional ethical permission (ORECNI, 08/NIR03/73) and sourced from the Belfast Health & Social Care Trust, ISU Abxis Co (Cepheid, Stretton, UK) and US Biomax Inc (Insight Biotechnology Ltd, Wembley, UK).

### Analysis of UCH-L1 expression and NSCLC patient survival in publicaly available datasets

Three relevant publicaly available lung cancer datasets (GSE13213, GSE3141 and GSE13213) which contained whole-genome profiles and associated patient outcome data were identified in the Gene Expression Omnibus (GEO) database repository. GSE13213 consisted of whole-genome expression profiles of 117 adenocarcinoma samples with the associated outcome data of "days survival". GSE3141 consisted of 111 primary lung tumour samples with associated survival data stated in "months survival" and GSE8894 contained gene expression profiles from primary tumours from 138 lung cancer patients with associated "recurrence free survival (months)" outcome data. Expression profiles for GSE13212 were generated using the Agilent-014850 Whole Human Genome Microarray 4 × 44 K G4112F platform which contains 1 probe for the UCH-L1 gene (A_23P132956). For both GSE3141 and GSE8894 datasets, gene expression profiles were generated using Affymetrix Human Genome U133 Plus 2.0 Array which contains 2 probesets for the UCH-L1 gene (1555834_at, 201387_s_at). The Series Matrix files were downloaded from GEO for all 3 datasets. Normalized expression data and associated outcome data were imported into the Partek Genomics Suite (Partek Inc, St Louis, MO). Patients were separated into quartiles based on expression levels of the UCH-L1 gene for each dataset. The survival times for each quartile were compared using Kaplan-Meier survival analysis and the log-rank test.

### Statistical Analysis

All experiments were carried out with a minimum of *n *= 3. Intergroup comparisons were made by Student's *t *test with *P *< 0.05 considered statistically significant.

## Results

### Expression of UCH-L1 in non-small cell lung carcinoma lines

**Figure 1:**
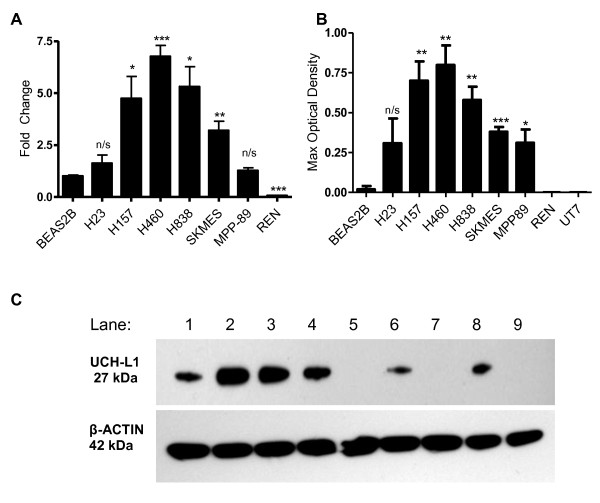
**UCH-L1 expression is higher in NSCLC cell lines than in normal lung cells**. **A**. Fold change of UCH-L1 mRNA in lung cancer cell lines compared to the normal lung cell line BEAS-2B (n = 3). **B**. Densitometry of the level of UCH-L1 protein detected by Western Blot relative to the level of β-actin detected (n = 3). **C**. Western Blot detection of UCH-L1 protein and β-actin loading control in different cell lines. Lanes as follows: 1 = H23, 2 = H157, 3 = H460, 4 = H838, 5 = BEAS-2B, 6 = MPP-89, 7 = REN, 8 = SKMES, 9 = UT-7.

**Figure 2:**
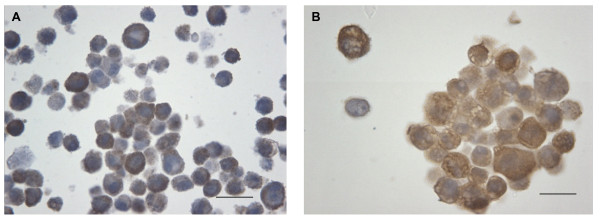
**Immunohistochemistry showing UCH-L1 positive cells in H157 and H838 cells**. Brown staining indicates the presence of UCH-L1 in H157 (**A**) and H838 (**B**) cells. (Scale bar is equivalent to 15 μm).

To identify a cell line model exhibiting high UCH-L1 expression that could be modulated for further investigations a range of human non-small cell lung carcinoma cell lines was surveyed for UCH-L1 expression by q-PCR and immunoblotting and compared to a normal lung cell line BEAS-2B (Figure 1). This revealed several cell lines (H157, H460 and H838) with high levels of *UCH-L1 *mRNA expression (Figure 1A). Interestingly, the cell lines with elevated *UCH-L1 *expression had differing origins; H460 is a large cell lung carcinoma while H157 is of squamous cell origin and H838 is an adenocarcinoma established from a metastatic lymph node. The level of UCH-L1 protein was found to reflect mRNA expression shown in Figure 1B &1C, with H157, H460 and H838 exhibiting abundant protein production. Sequencing the UCH-L1 gene in these different cell lines failed to detect any mutations. Cell blocks of H157 and H838 cells were also stained by immunohistochemistry for UCH-L1 expression and both stained positive for the protein (Figure 2A and 2B).

### Silencing of UCH-L1 expression in the H838 and H157 cell lines

**Figure 3:**
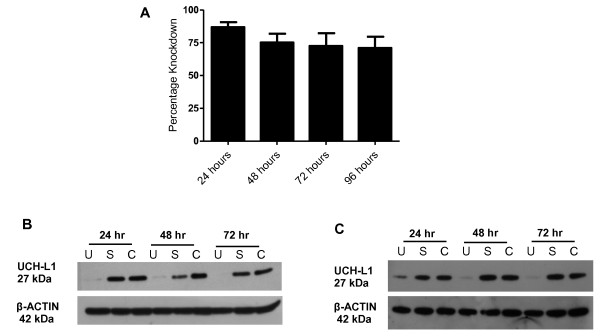
**Knockdown of UCH-L1 in H838 and H157 cells by siRNA**. A. Percentage knockdown of UCH-L1 mRNA in H838 cells at 24 hr, 48 hr, 72 hr and 96 hr post-transfection compared to time-matched control. **B & C**. Immunoblot detection of UCH-L1 protein expression at 24 hr, 48 hr and 72 hr post-transfection in H838 cells (B) and H157 cells (C). (U = UCH-L1 siRNA, S = Scrambled siRNA, C = Untreated control).

To establish if elevated UCH-L1 levels contribute to lung carcinogenesis, expression in H157 and H838 cells was silenced using siRNA and any subsequent phenotypic changes were investigated. *UCH-L1 *mRNA was substantially down-regulated in H838 cells at 24 hr post-transfection and remained decreased at 96 hr post-transfection (Figure 3A). Immunoblotting confirmed UCH-L1 protein was significantly reduced at 24 hr post-transfection and by 72 hr the protein was undetectable in both H838 cells (Figure 3B) and H157 cells (Figure 3C).

### UCH-L1 supports cell survival in H838 cells

**Figure 4:**
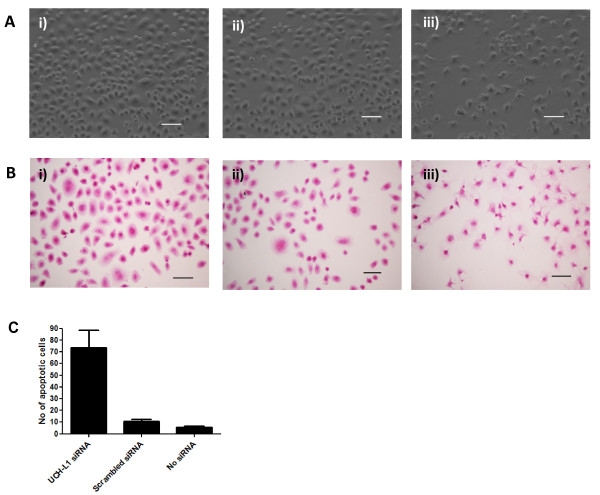
**Reduced UCH-L1 expression alters morphology of H838 cells and increases the number of apoptotic cells**. **A**. Phase-contrast microscopy photographs of i) non-transfected H838 cells; ii) scrambled siRNA-treated H838 cells; iii) UCH-L1 siRNA-treated H838 cells. **B**. H & E staining of i) non-transfected H838 cells; ii) scrambled siRNA-treated H838 cells; iii) UCH-L1 siRNA-treated H838 cells. (Scale bar is equivalent to 15 μm). **C**. Number of apoptotic cells counted in 20 fields of H&E stained slides at 250× magnification.

Assessment of H838 and H157 cells exhibiting reduced UCH-L1 protein levels by phase-contrast microscopy revealed morphological changes in the UCH-L1 siRNA-treated H838 cells compared to scrambled siRNA- treated and untreated control cells, whereas no difference was observed between UCH-L1 siRNA-treated H157 cells and control H157 cells. Normally the parental H838 cells were rounded in shape and uniform in size, but cells with reduced UCH-L1 expression were irregular in shape, variable in size, and present at a much lower density. H838 cells with low levels of UCH-L1 were also less flattened to the surface, possibly signifying they were becoming detached, a characteristic of apoptotic cells (Figure 4A). Therefore untreated and treated H838 cells were stained with H&E to compare the number of apoptotic cells. Definite apoptotic changes were observed in the UCH-L1 siRNA-treated cells (Figure 4B). To quantify the differences in apoptosis between the siRNA-treated and untreated cells, the number of apoptotic cells as characterised by fragmentation of the nucleus or breakdown of the nuclear envelope were counted in 20 fields of view at 250× magnification. A large increase in the number of apoptotic cells was observed in H838 cells with reduced UCH-L1 expression, which was statistically significant with a p-value of < 0.01 (Figure 4C).

**Figure 5:**
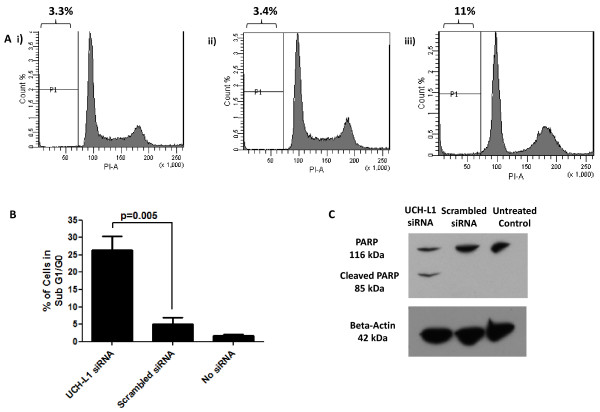
**UCH-L1 expression in H838 cells confers apoptotic resistance measured by flow cytometry and PARP cleavage**. A. Comparison of cell cycle analysis of propidium iodide stained untreated H838 cells (Panel i), scrambled siRNA-treated H838 cells (Panel ii) and H838 cells treated with UCH-L1 siRNA (Panel iii). The percentage of cells in sub G1/G0 are shown above each panel. **B**. The percentage of cells in sub G1/G0 phase of the cell cycle in each treatment group for 3 independent experiments are shown graphically. **C**. Immunoblot showing PARP cleavage in siRNA-treated and parental H838 cells.

Since apoptosis results in an increased number of cells in the sub G1/G0 phase of the cell cycle, flow cytometry was used to quantify this specific population of cells. H838 cells with reduced UCH-L1 were observed to have a greater proportion, around 30%, of cells in sub G1/G0 phase which was statistically significant, and there was an overall decrease in the total cell population which correlates with an increased rate of apoptosis (Figure 5A &5B). To further confirm apoptosis was present, PARP cleavage was measured by immunoblotting. Cleavage of the PARP protein into two fragments, an early indicator of apoptosis, was only apparent in H838 cells post UCH-L1 siRNA knock-down (Figure 5C). Studying cell proliferation using CyQUANT^® ^assays at two different time points post-transfection indicated that loss of UCH-L1 expression did not affect cell proliferation (Additional File 1). In contrast, H157 cells did not exhibit apoptotic features when UCH-L1 expression was reduced and no reduction in proliferation was observed as measured by Ki67 staining (data not shown).

### UCH-L1 promotes cell migration in H157 cells

**Figure 6:**
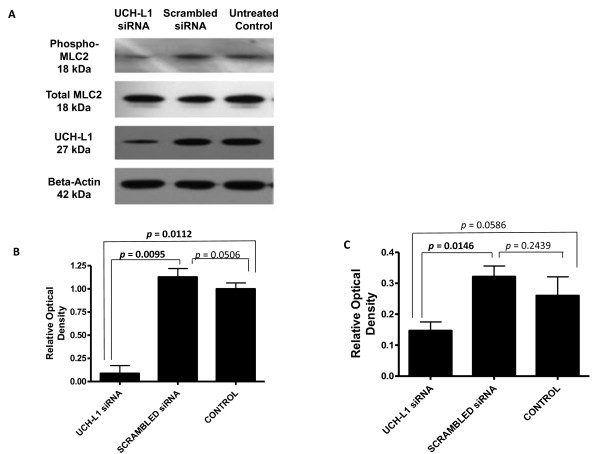
**Lower levels of UCH-L1 decrease phosphorylation of MLC2 in H157 cells**. **A**. Immunoblot of pMLC-2 protein, total MLC2, UCH-L1 knockdown and β-actin loading control in H157 cells post siRNA treatment. **B**. Densitometry analysis for 3 sets of blots exhibiting UCH-L1 protein level in untreated H157 cells and cells treated with either scrambled siRNA or UCH-L1 siRNA. UCH-L1 protein levels in H157 cells were normalized to β-actin. **C**. Densitometry analysis for 3 sets of blots exhibiting MLC2 phosphorylation in untreated H157 cells and cells treated with either scrambled siRNA or UCH-L1 siRNA. Phospho-MLC2 protein levels in H157 cells were normalized to β-actin.

Although loss of UCH-L1 expression did not affect cell viability in H157 cells, it could influence the metastatic process since previous studies have implicated UCH-L1 in metastasis of tumour cells [17,26,30]. Cell migration assays can be used as an indicator of metastatic potential, therefore the protein level of phosphorylated myosin light chain (MLC2), a surrogate marker for migratory capacity, was measured by immunoblotting. A reduction in phosphorylated MLC2 in H157 cells post siRNA transfection was detected (Figure 6A), whereas total MLC2 levels remained constant (Figure 6A). Statistical analysis showed the level of phospho-MLC2 was significantly reduced in the siRNA treated cells compared to those treated with scrambled siRNA but less so when compared to the untreated control H157 cells (Figure 6B and 6C). It was not possible to analyze the migratory capacity of H838 cells as the cells following UCH-L1 knockdown were of too poor a quality to give reproducible results.

### Relevance of UCH-L1 over-expression in NSCLC patient tumour samples

**Figure 7:**
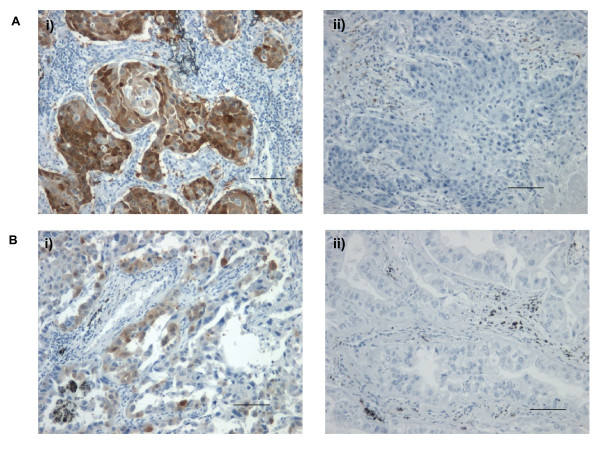
**UCH-L1 expression in adenocarcinoma and squamous cell carcinoma**. A. Squamous cell carcinoma stained positive (i) and negative (ii) for UCH-L1. **B**. Adenocarcinoma positive (i) and negative (ii) for UCH-L1 expression. Brown staining indicates the presence UCH-L1. (Scale bar is equivalent to 25 μm).

To establish if UCH-L1 is consistently overexpressed in NSCLC tumour samples 140 cases (85 squamous cell carcinomas and 55 adenocarcinomas) were screened for UCH-L1 positivity by immunohistochemistry (Figure 7A and 7B). Overexpression of UCH-L1 was detected in 47 cases (34.3%) and among these positive cases 37 were squamous cell carcinoma and 10 cases were adenocarcinoma hence UCH-L1 was correlated with histological type (r = 0.262).

### UCH-L1 expression does not correlate with long term survival

**Figure 8:**
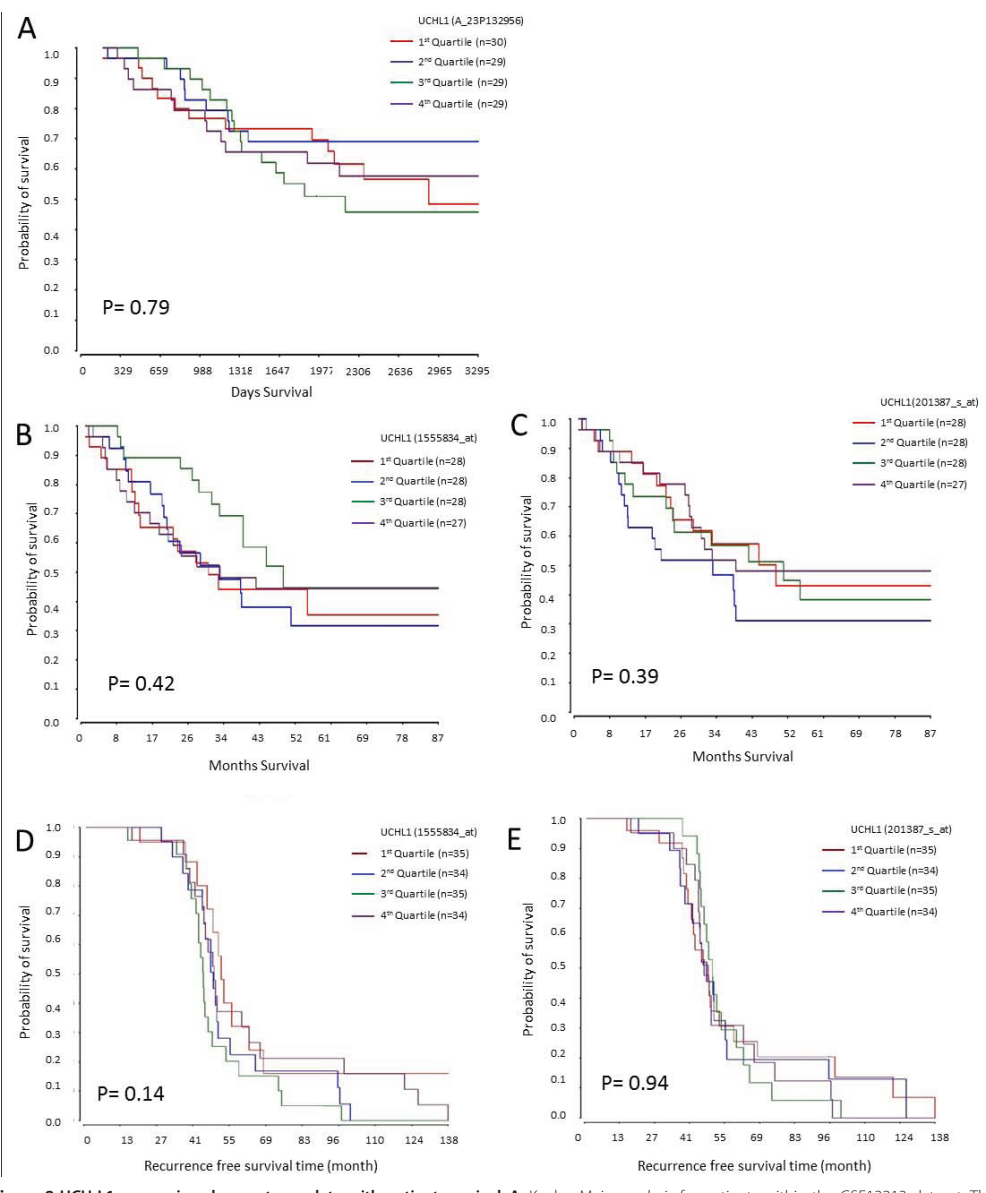
**UCH-L1 expression does not correlate with patient survival**. **A**. Kaplan-Meier analysis for patients within the GSE13213 dataset. The UCH-L1 gene was represented by a single probeset (A-23P132956). The time variable was "days survival" and the event variable was "alive or dead". **B &C. Kaplan-Meier analysis for patients within the GSE3141 dataset**. The time variable stated was "months survival" and the event variable was "dead or alive". The UCH-L1 gene was represented by 2 separate probesets (1555834_at and 201387_s_at). Individual Kaplan-Meier plots were generated for each of the probesets (B-probeset 1555834_at and C-probeset 201387_s_at). **D & E. Kaplan-Meier analysis for patients within the GSE8894 dataset**. The time variable used was "recurrence free survival" and the event variable was "recurrence or non-recurrence". The UCH-L1 gene was represented by 2 separate probesets (1555834_at and 201387_s_at). Individual Kaplan-Meier plots were generated for each of the probesets (D-probeset 1555834_at and E-probeset 201387_s_at).

To investigate if the potential oncogenic role of UCH-L1 observed in the cell line model is reflected in patients, Kaplan-Meier plots were generated for NSCLC patients based on UCH-L1 expression. To do this three microarray-based gene expression studies with associated patient outcome data (accession numbers GSE13213, GSE8894 and GSE3141) were identified that were available from the NCBI's Gene Expression Ombnibus (GEO). Normalized microarray data and phenotype data were downloaded and samples were separated into quartiles according to UCH-L1 expression levels. Kaplan-Meier survival analysis, including the log-rank test, was performed on each of the quartiles. No significant difference in survival was observed between the quartiles for all three datasets (Figure 8). Kaplan-Meier survival analysis was also performed on patients separated into above and below the median and on the upper and lower quartiles for UCH-L1 expression. In all 3 datasets no significant difference was observed in any of the comparisons (Additional files 2, 3 and 4).

## Discussion

The present study indicates that UCH-L1 is highly expressed in lung squamous cell carcinoma, and NSCLC cell line studies show that increased UCH-L1 expression causes apoptotic resistance in H838 adenocarcinoma cells and a greater capacity for cell migration in the H157 squamous cell carcinoma cell line. However, despite the oncogenic effects of UCH-L1 observed in NSCLC cell lines, its expression does not appear to affect patient survival in NSCLC.

Our findings reveal that 4 of 5 NSCLC cell lines analyzed exhibit statistically significant increases in wild type UCH-L1 expression when compared to the normal lung cell line and approximately one third of 140 NSCLC samples (stage II/III) stain positive for UCH-L1 by immunohistochemistry. This confirms previous reports that UCH-L1 is highly expressed in NSCLC cell lines and primary tumours. UCH-L1 staining also correlates with histology as squamous cell carcinomas express the protein more frequently than adenocarcinomas. Although Sasaki et al [34] found no such association, our results are in agreement with a previous study in which 72% squamous cell carcinoma tumours were positive for UCH-L1 in comparison to 41% in the adenocarcinoma subset [24].

The functional role of UCH-L1 in lung tumourigenesis however remains elusive, therefore following confirmation of high UCH-L1 expression we examined the phenotypic effects in NSCLC cell lines. The expression of UCH-L1 was reduced using siRNA in both squamous cell carcinoma (H157) and adenocarcinoma (H838) cell lines. Knockdown of UCH-L1 in H838 cells shows morphological differences indicative of apoptosis and cell death was confirmed by H&E staining, cell cycle analysis and the presence of PARP cleavage. Although other studies have not examined the effect of UCH-L1 specifically in H838 cells, UCH-L1 has been associated with apoptosis in several cases. In neuronal cells and testicular germ cells UCH-L1 is viewed as an apoptosis-promoting protein due to its role in balancing the levels of pro-apoptotic and anti-apoptotic proteins [9,11,12]. In contrast, the current investigation shows that UCH-L1 increases apoptotic resistance, confirming a number of recent reports [15,38]. Treatment of neuroblastoma cells with an UCH-L1 inhibitor was shown to cause apoptosis, mediated through decreased activity of the proteasome and accumulation of highly ubiquitinated proteins. This caused endoplasmic reticulum stress in the neuroblastoma cells which eventually led to the initiation of cell death [38]. Likewise, the up-regulation of UCH-L1 in human hepatoma cells following low dose UV irradiation was reported to be involved in the regulation of cell death by inhibition of p53-mediated apoptosis; hence in both these cases UCH-L1 was demonstrated to be an "apoptosis-evading protein" [39], as in the present study.

In contrast to H838 cells, our study reveals UCH-L1 knockdown causes no difference in morphology, apoptosis or proliferation in H157 cells but does reduce the capacity for cell migration. MLC2, a protein responsible for cell movement, is phosphorylated during cell invasion [40]. In this present study it was shown that reduced expression of UCH-L1 in H157 cells led to decreased phosphorylation of MLC2, suggesting that UCH-L1 may be involved in tumour cell migration. This challenges the findings of a recent study in which treatment of H157 cells with UCH-L1 siRNA resulted in increased apoptosis and inhibition of proliferation [33]. Conversely, we observed no morphological differences in H157 cells and no effect on proliferation (measured by Ki67 staining) when UCH-L1 expression was knocked down. In support of our observations, Kim et al [32] demonstrated no effect on any phase of the cell cycle but UCH-L1 expression increased invasive capacity of H157 cells as measured by both Matrigel invasion assay and wound healing assays. However, Kim et al [32] used a different system that utilized an inducible lentiviral vector expressing shRNA rather than oligonucleotide transfection of siRNA.

Taken together our results suggest that in addition to the correlation of UCH-L1 expression with histological type, the functional effects of UCH-L1 on NSCLC cells may also be subtype-dependent. Analysis of UCH-L1 in the large cell carcinoma cell line H1299 presents yet another different role for this protein in NSCLC since UCH-L1 was found to be antiproliferative in this case and the authors concluded that it is expressed as a response to tumour growth [41].

Our cell line studies suggest that UCH-L1 expression may be important in the pathogenesis of lung cancer. *In vivo *studies of UCH-L1 expression in the lung have also demonstrated a role for UCH-L1 in lung carcinogenesis in two separate reports. When BALB/C nude mice were injected with UCH-L1-expressing metastatic melanoma cells, black melanoma colonies were generated in the lungs but when melanoma cells treated with UCH-L1 siRNA were introduced there was a significant decrease in the number of metastatic lung colonies [32]. Additionally, Hussain et al [3] demonstrated the spontaneous development of lung tumours in an UCH-L1-overexpressing transgenic mouse model.

To assess the relevance of UCH-L1 in patient samples we looked at whether high or low UCH-L1 expression resulted in any difference in survival status of NSCLC patients. Despite the evidence supporting a role for UCH-L1 in lung carcinogenesis in the cell line study, UCH-L1 status was not significantly associated with patient outcome. This was particularly surprising considering high UCH-L1 expression in NSCLC was previously correlated with an advanced tumour stage. However, Sasaki et al [34] also failed to find a link with survival. Therefore, although cell line models seem to indicate an oncogenic role of UCH-L1 this does not appear to translate into patient samples.

## Conclusions

In conclusion, this study shows the expression of UCH-L1 in NSCLC is variable and dependent on histological type. In cell line models UCH-L1 appears to have an oncogenic role in NSCLC leading to increased apoptotic resistance in H838 adenocarcinoma cells and a greater capacity for migration in the squamous cell carcinoma cell line (H157).

Despite the promising observations in the NSCLC cell lines following UCH-L1 knockdown, translation to the clinical setting did not indicate any correlation with patient survival. Thus caution is required when using UCH-L1 as a prognostic marker in isolation for advanced stage and metastasis in lung carcinoma as other factors may be involved. Clearly further investigation would be required to establish whether UCH-L1 is part of a pathway that influences prognosis in lung cancer.

## Competing interests

The authors declare that they have no competing interests.

## Authors' contributions

KSO performed siRNA knockdown, apoptosis and metastatic potential assays, and prepared the manuscript. ZS conceived the study and designed the siRNA knockdown and apoptosis assays. WMB generated Kaplan-Meier curves, analyzed patient survival data, and prepared the manuscript. KOH generated Kaplan-Meier curves, analyzed patient survival data, reviewed all statistics and reviewed the manuscript. TRL conceived the study, participated in the design and prepared the manuscript. PM performed immunohistochemical analysis of tumours microarrays and prepared the manuscript. MJP conceived the study, participated in the design and prepared the manuscript. All authors read and approved the final manuscript.

## Supplementary Material

Additional file 1 **Loss of UCH-L1 expression did not affect cell proliferation of H838 cells**. CyQUANT^® ^assays were performed at two different time points of 24 and 48 hr post-transfection with UCH-L1 siRNA in H838 cells. The results from 3 experiments are shown graphically. Statistical analysis showed no significant difference between UCH-L1 siRNA-treated and controls. Click here for file

Additional file 2 **Kaplan-Meier analysis in the GSE13213 dataset based on UCH-L1 expression**. **A**. Kaplan-Meier analysis for patients separated into above and below the median of UCH-L1 expression in the GSE13213 dataset. B Kaplan-Meier analysis for patients separated into quartiles based on UCH-L1 expression. The first and fourth quartiles are included in the graph. The UCH-L1 gene is represented by a single probe (A-23P132956). Click here for file

Additional file 3 **Kaplan-Meier analysis in the GSE3141 dataset based on UCH-L1 expression represented by probesets 1555834_at and 201387_s_at**. **A**. Kaplan-Meier analysis for patients separated into above and below the median expression of UCH-L1 based on probeset 1555834_at signal intensities. **B**. Kaplan-Meier analysis for patients separated into quartiles based on UCH-L1 expression represented by probeset 1555834_at. The first and fourth quartiles are included in the graph. **C**. Kaplan-Meier analysis for patients separated into above and below the median expression of UCH-L1 based on probeset 201387_s_at signal intensities. **D**. Kaplan-Meier analysis for patients separated into quartiles based on UCH-L1 expression represented by 201387_s_at. The first and fourth quartiles are included in the graph. Click here for file

Additional file 4 **Kaplan-Meier analysis in the GSE8894 dataset based on UCHL-1 expression represented by 2 probesets 1555834_at and 201387_s_at**. **A**. Kaplan-Meier analysis for patients separated into above and below the median expression of UCH-L1 based on probeset 1555834_at signal intensities. **B**. Kaplan-Meier analysis for patients separated into quartiles based on UCH-L1 expression represented by probeset 1555834_at. The first and fourth quartiles are included in the graph. **C**. Kaplan-Meier analysis for patients separated into above and below the median expression of UCH-L1 based on probeset 201387_s_at signal intensities. **D**. Kaplan-Meier analysis for patients separated into quartiles based on UCH-L1 expression represented by 201387_s_at. The first and fourth quartiles are included in the graph. Click here for file
