# Supplementary figures and images for: Potential prognostic marker ubiquitin carboxyl-terminal hydrolase-L1 does not predict patient survival in non-small cell lung carcinoma
Source: J Exp Clin Cancer Res. 2011 Aug 30;30(1):79. doi: 10.1186/1756-9966-30-79 (PMC3180428; doi:10.1186/1756-9966-30-79)

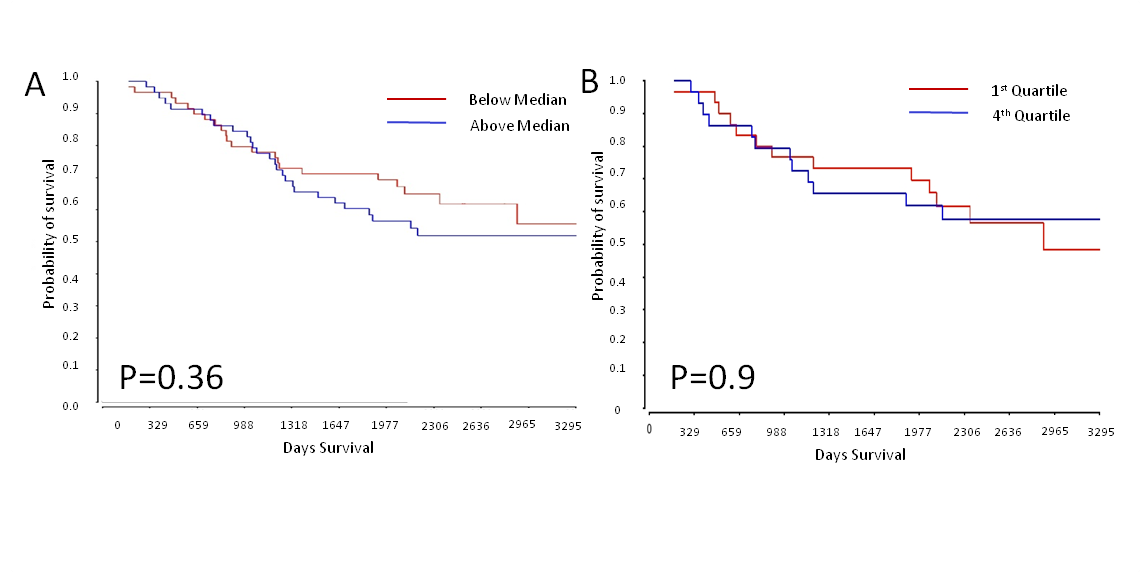

Supplement: Additional file 2 — Kaplan-Meier analysis in the GSE13213 dataset based on UCH-L1 expression. A. Kaplan-Meier analysis for patients separated into above and below the median of UCH-L1 expression in the GSE13213 dataset. B Kaplan-Meier analysis for patients separated into quartiles based on UCH-L1 expression. The first and fourth quartiles are included in the graph. The UCH-L1 gene is represented by a single probe (A-23P132956). [file 1756-9966-30-79-S2.TIFF]

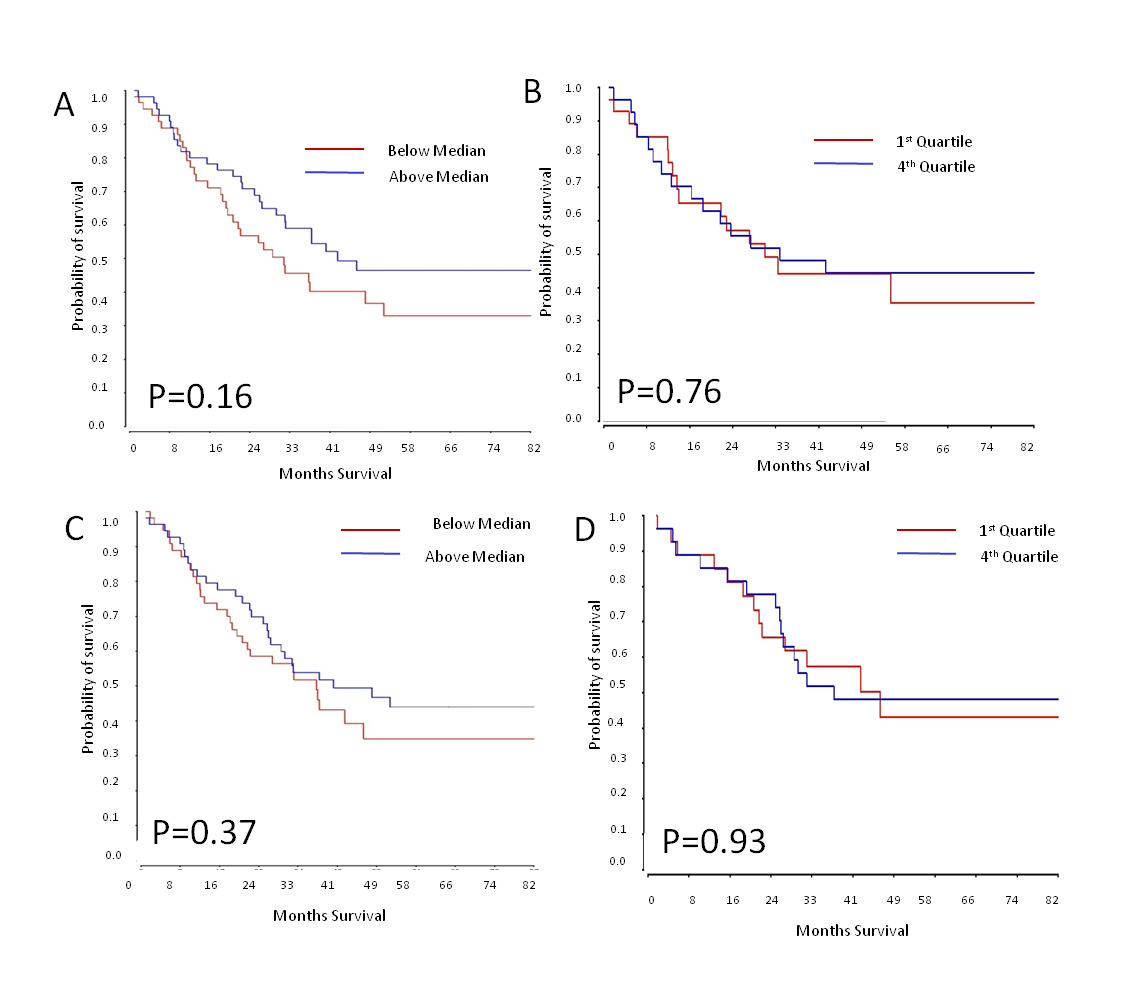

Supplement: Additional file 3 — Kaplan-Meier analysis in the GSE3141 dataset based on UCH-L1 expression represented by probesets 1555834_at and 201387_s_at. A. Kaplan-Meier analysis for patients separated into above and below the median expression of UCH-L1 based on probeset 1555834_at signal intensities. B. Kaplan-Meier analysis for patients separated into quartiles based on UCH-L1 expression represented by probeset 1555834_at. The first and fourth quartiles are included in the graph. C. Kaplan-Meier analysis for patients separated into above and below the median expression of UCH-L1 based on probeset 201387_s_at signal intensities. D. Kaplan-Meier analysis for patients separated into quartiles based on UCH-L1 expression represented by 201387_s_at. The first and fourth quartiles are included in the graph. [file 1756-9966-30-79-S3.TIFF]

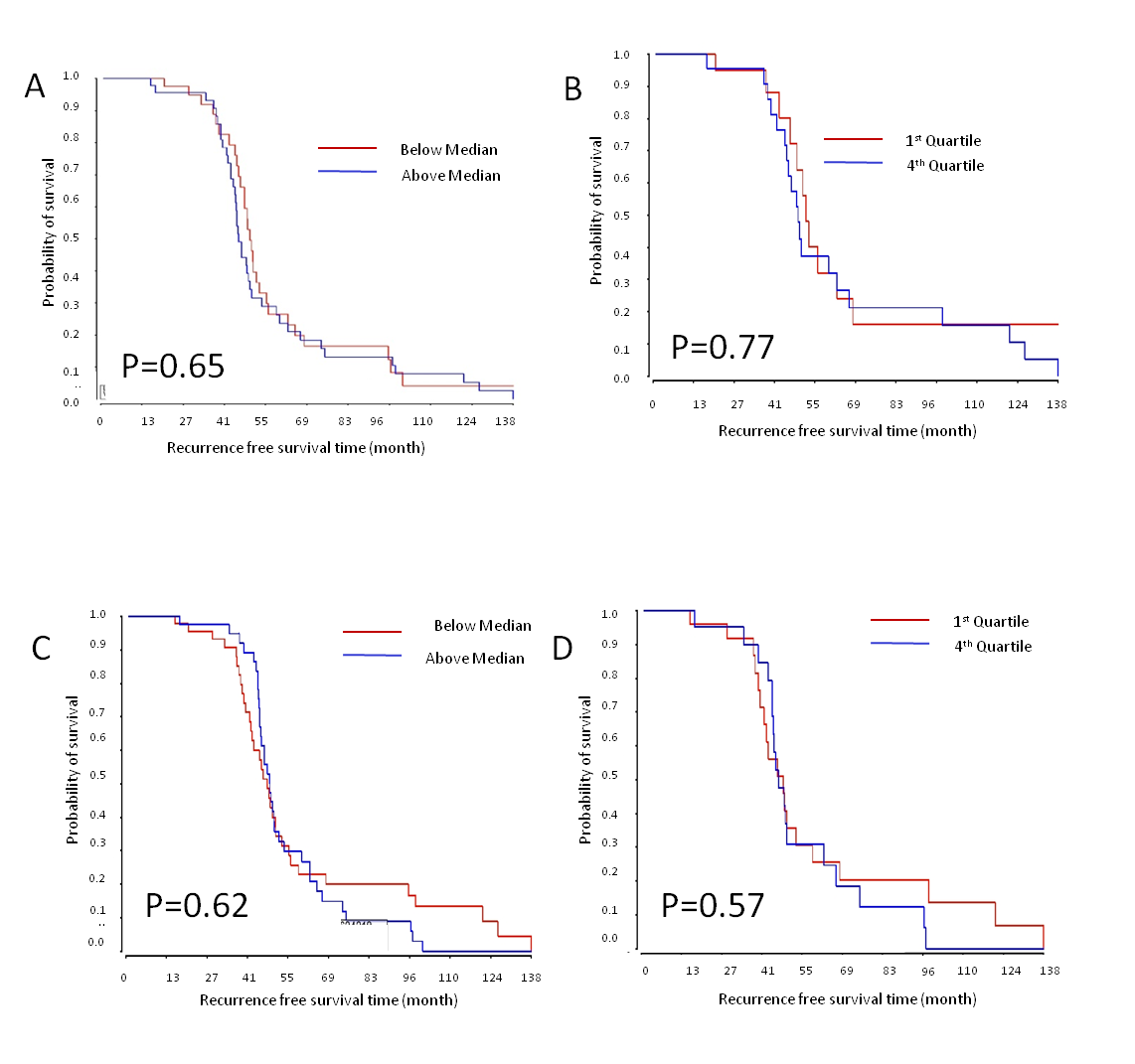

Supplement: Additional file 4 — Kaplan-Meier analysis in the GSE8894 dataset based on UCHL-1 expression represented by 2 probesets 1555834_at and 201387_s_at. A. Kaplan-Meier analysis for patients separated into above and below the median expression of UCH-L1 based on probeset 1555834_at signal intensities. B. Kaplan-Meier analysis for patients separated into quartiles based on UCH-L1 expression represented by probeset 1555834_at. The first and fourth quartiles are included in the graph. C. Kaplan-Meier analysis for patients separated into above and below the median expression of UCH-L1 based on probeset 201387_s_at signal intensities. D. Kaplan-Meier analysis for patients separated into quartiles based on UCH-L1 expression represented by 201387_s_at. The first and fourth quartiles are included in the graph. [file 1756-9966-30-79-S4.TIFF]
